# The Role of Antifibrotic Therapy in Pulmonary Fibrosis and Lung Cancer: A Multicenter Retrospective Analysis

**DOI:** 10.3390/biomedicines13092310

**Published:** 2025-09-21

**Authors:** Francesco Rocco Bertuccio, Nicola Baio, Fabio Perrotta, Donato Lacedonia, Vito D’Agnano, Andrea Bianco, Giulia Scioscia, Pasquale Tondo, Maria Pia Foschino Barbaro, Chandra Bortolotto, Angelo Guido Corsico, Giulia Maria Stella

**Affiliations:** 1 Department of Internal Medicine and Medical Therapeutics, University of Pavia Medical School, 27100 Pavia, Italy; francesco.bertuccio01@gmail.com (F.R.B.); angelo.corsico@unipv.it (A.G.C.); 2 Cardiothoracic and Vascular Department, Unit of Respiratory Diseases, Istituto di Ricerca e Cura a Carattere Scientifico (IRCCS) Policlinico San Matteo, 27100 Pavia, Italy; 3 Ospedale Maggiore, Unit of Pneumology, Azienda Socio-Sanitaria Territoriale (ASST) Crema, 26013 Crema, Italy; nicola.baio01@universitadipavia.it; 4 Department of Translational Medical Sciences, University of Campania “L. Vanvitelli”, 80100 Napoli, Italy; fabio.perrotta@unicampania.it (F.P.); vito.dagnano@studenti.unicampania.it (V.D.); andrea.bianco@unicampania.it (A.B.); 5 Department of Medical and Surgical Sciences, University of Foggia, 71121 Foggia, Italy; donato.lacedonia@unifg.it (D.L.); giulia.scioscia@unifg.it (G.S.); pasquale.tondo@unifg.it (P.T.); mariapia.foschino@unifg.it (M.P.F.B.); 6 Department of Specialist Medicine, Institute of Respiratory Diseases, University-Hospital Polyclinic “Riuniti”, 71121 Foggia, Italy; 7 Diagnostic Imaging and Radiotherapy Unit, Department of Clinical, Surgical, Diagnostic and Pediatric Sciences, University of Pavia Medical School, 27100 Pavia, Italy; chandra.bortolotto@unipv.it; 8 Radiology Institute, Fondazione Istituto di Ricovero e Cura a Carattere Scientifico (IRCCS) Policlinico San Matteo, 27100 Pavia, Italy

**Keywords:** pulmonary fibrosis, interstitial lung disease, lung cancer, antifibrotics, nintedanib, pirfenidone, acute exacerbation, PD-L1, immunotherapy

## Abstract

**Background**: Patients with fibrotic interstitial lung disease (ILD) are at increased risk of lung cancer, yet the impact of antifibrotic therapy on oncologic outcomes remains unclear. Objective: This study aimed to explore associations between antifibrotic therapy and overall survival (OS) and acute exacerbations of ILD (AE-ILD) in patients with fibrotic ILD who develop lung cancer. **Methods**: We retrospectively analyzed 61 patients from multiple Italian centers: 35 received antifibrotic therapy (pirfenidone or nintedanib) and 26 did not. Outcomes included OS from cancer diagnosis and post-treatment AE-ILD. **Results**: Mean OS was 17.9 months in the antifibrotic group and 33.2 months in the non-antifibrotic group; no adjusted survival analyses were possible due to missing censoring data, and these descriptive values should not be overinterpreted. AE-ILD occurred in 11.4% of antifibrotic-treated patients and 11.5% of those without antifibrotics. PD-L1 expression was detected in 24.1% vs. 21.8% of tumors in the two groups, and autoantibody positivity was observed in 22.8% vs. 30.7%, respectively, reflecting differences in ILD subtypes. **Conclusions**: In this heterogeneous real-world cohort, antifibrotic therapy was not associated with increased AE-ILD risk, and descriptive OS comparisons showed no clear survival advantage. These exploratory findings warrant confirmation in larger, prospective studies.

## 1. Introduction

Fibrotic interstitial lung disease (ILD) is associated with an increased incidence of lung cancer, likely reflecting shared risk factors, chronic epithelial injury, and pro-fibrotic/pro-oncogenic signaling. Despite the growing use of antifibrotic agents, their relationship with oncologic outcomes remains uncertain.

Evidence on whether antifibrotics influence overall survival (OS) or the risk of treatment-related acute exacerbations (AE-ILD) is limited and heterogeneous, with small samples and variable phenotypes complicating inference. Moreover, the role of PD-L1 expression and autoimmunity in this population is poorly characterized.

Aim: The aim of this multicenter retrospective study was to explore associations between antifibrotic therapy and (i) OS after cancer diagnosis and (ii) post-treatment AE-ILD in patients with fibrotic ILD who develop lung cancer, and to provide hypothesis-generating observations regarding PD-L1 and autoimmune features.

### 1.1. Lung Cancer and Interstitial Lung Disease (ILD): Pathogenetic Mechanisms

Chronic inflammatory injury at the alveolar level (though triggered by distinct etiologies depending on the underlying disease and modulated in severity by genetic susceptibility) is a hallmark of both fibrosing interstitial lung diseases (ILDs) and lung cancer. This persistent inflammation promotes the release of cytokines and chemokines that establish a pro-inflammatory microenvironment. As a result, a cascade of aberrant tissue repair and remodeling processes is initiated, ultimately leading to the excessive deposition of extracellular matrix (ECM) in the interstitium and the development of pulmonary fibrosis [1].

Chronic inflammation due to smoking, emphysema, or established fibrosis significantly increases the risk of malignant transformation. Injured epithelial cells release pro-inflammatory mediators, resulting in elevated levels of interleukin-6 (IL-6) and C-reactive protein (CRP). This inflammatory milieu triggers immune responses that damage alveolar structures and promote epithelial–mesenchymal transition (EMT), a key event that facilitates carcinogenesis.

In idiopathic pulmonary fibrosis (IPF), alveolar epithelial cell injury leads to the release of interleukin-1 (IL-1), tumor necrosis factor-alpha (TNF-α), and chemokine CCL2. These mediators activate neighboring cells and recruit inflammatory cells from the circulation. A wide range of immune cells, including macrophages and predominantly Th2 lymphocytes, participate in this process by releasing pro-inflammatory cytokines such as IL-4 and IL-13. Both epithelial and endothelial cells contribute to fibrogenesis by secreting profibrotic mediators, among which transforming growth factor-beta (TGF-β) plays a pivotal role [1,2,3].

This chronic inflammatory state and ECM remodeling establish a microenvironment conducive to malignant transformation. Several tyrosine kinase-dependent signaling pathways, known to regulate cellular proliferation, differentiation, adhesion, and motility, are implicated in both pulmonary fibrosis and lung cancer [4].

Programmed death-ligand 1 (PD-L1) has been found to be overexpressed as a membrane-bound protein in both lung cancer cells and fibrotic lung tissue. By interacting with membrane receptors on T lymphocytes, PD-L1 suppresses immune responses and facilitates unchecked cellular proliferation [5,6].

Epigenetic alterations, such as hypermethylation of tumor suppressor genes and hypomethylation of oncogenes, have been observed in the pathogenesis of both idiopathic pulmonary fibrosis (IPF) and lung cancer [1]. Furthermore, common molecular alterations include TP53 mutations, downregulation of PTEN, and mutations in telomerase-related genes, such as TERC and TERT, all of which are shared between IPF and non-small-cell lung cancer (NSCLC) [7].

It is the synergistic interplay of these molecular and cellular mechanisms (albeit with disease-specific variations) that explains the increased predisposition in fibrotic ILDs not only to persistent and progressive fibrosis but also to malignant transformation of the affected cells [8].

This complexity underscores the fact that multiple pathogenic pathways are involved, suggesting numerous potential therapeutic targets. However, it also highlights a major challenge: current antifibrotic agents such as nintedanib and pirfenidone, which act primarily on single molecular targets, are likely insufficient to fully halt the degenerative process.

### 1.2. Incidence and Survival of Lung Cancer in ILDs

Depending on the underlying ILD and the study considered, the most common histological subtype may be either adenocarcinoma or squamous cell carcinoma (SCC) [9].

In idiopathic pulmonary fibrosis (IPF), SCC is more frequently observed. The incidence of lung cancer in patients with IPF is approximately 3.5 to 5 times higher than in individuals without IPF. The reported prevalence of lung cancer in IPF ranges from 2.7% to 48%, significantly higher than in the general population [10]. Lung cancer typically develops 12–36 months after the diagnosis of IPF. The 5-year survival rate after cancer diagnosis is 14.5% in patients with IPF, compared to 30.1% in those without IPF [11].

In connective tissue disease-associated ILDs (CTD-ILDs), the risk of lung cancer is twice as high compared to other non-CTD ILDs. Among CTDs, systemic sclerosis (SSc) and myositis show the strongest association with cancer development, followed by rheumatoid arthritis (RA). In such cases, the risk of neoplastic transformation can be up to five times higher than in the general population [3,12]. In fibrotic hypersensitivity pneumonitis (fHP), the estimated prevalence of lung cancer exceeds 10%, with SCC again being the most frequently reported histotype [13].

A meta-analysis involving 620 patients with combined pulmonary fibrosis and emphysema (CPFE) revealed that lung cancer tends to affect younger, male, smoking individuals, with a sevenfold increased risk compared to those without CPFE [10,14,15].

### 1.3. Antifibrotic Therapy in Lung Cancer and the Role of PD-L1

Nintedanib and Pirfenidone, introduced into clinical practice over a decade ago, are two antifibrotic agents with distinct mechanisms of action, both capable of slowing the progression of pulmonary fibrosis. Recent evidence suggests that these drugs may also exert antitumor effects, although the molecular basis underlying this dual action remains to be fully elucidated [7].

### 1.4. Pirfenidone

Pirfenidone is an inhibitor of the TGF-β signaling pathway, exerting antifibrotic effects through suppression of fibroblast proliferation and collagen synthesis. The pivotal clinical trials leading to its approval demonstrated a reduction in mortality and in the rate of FVC decline in patients with IPF [2]. By inhibiting TGF-β, pirfenidone can reduce EMT and consequently suppress tumor progression. A study by Kurimoto et al. demonstrated that pirfenidone, similarly to nintedanib, inhibits EMT in lung adenocarcinoma models and reduces key features of malignancy [16].

The same study showed that PD-L1 expression in adenocarcinoma cells is upregulated by EMT induction and downregulated by its inhibition, highlighting a mechanistic link between EMT and tumor immune evasion. PD-L1. Beyond its role in lung cancer, PD-L1 has also been implicated in pulmonary fibrosis: in vitro studies show that PD-L1 inhibition can reduce TGF-β-mediated ECM production. Furthermore, elevated PD-L1 expression has been reported in the peripheral blood and pulmonary epithelial cells of patients with fibrotic lung disease, although the clinical significance of these findings requires further investigation. These data raise the intriguing hypothesis that the PD-1/PD-L1 axis could become a therapeutic target in fibrotic lung diseases [16,17].

A complex study by Shuling Zhang et al. demonstrated that pirfenidone inhibits the proliferation of specific metastatic NSCLC cell lines—particularly adenocarcinoma subtypes—but not squamous cell carcinoma, suggesting a selective effect mediated by specific metabolic pathways [18].

Additional studies have confirmed synergistic effects of pirfenidone with both cisplatin and carboplatin [19,20]. Given the drug’s dose-dependent efficacy and systemic toxicity, Vineela Parvathaneni explored a liposomal inhaled formulation of pirfenidone, which showed promising results in preclinical models [21].

### 1.5. Nintedanib

Nintedanib is a multitarget intracellular tyrosine kinase inhibitor that blocks signaling through VEGFR, FGFR, and PDGFR, thereby inhibiting fibroblast activation and angiogenesis. Initially developed as an antiangiogenic agent in oncology, nintedanib was subsequently approved for the treatment of IPF and, more recently, for progressive pulmonary fibrosis (PPF) of non-IPF origin based on the positive outcomes of the INBUILD trial [22,23].

A study by Otsubo et al. demonstrated that the addition of nintedanib to chemotherapy in patients with advanced adenocarcinoma and IPF increased progression-free survival, although no impact on overall survival was observed [24]. Notably, in carefully selected patients (ECOG PS 0–1 and GAP stage I–II), the incidence of acute exacerbations did not significantly differ between those treated with chemotherapy alone and those receiving the combination regimen [25,26].

Preclinical models have explored the synergistic potential of nintedanib with immune checkpoint inhibitors (ICIs). Jingyao Tu et al. showed that the combination of nintedanib with anti-PD-1/PD-L1 therapies enhanced immune cell infiltration within the tumor microenvironment and normalized tumor angiogenesis. In addition, increased expression of PD-L1 and MHC class I was observed, potentially improving the efficacy of ICIs and preventing the development of resistance [27].

While these findings are encouraging, they are largely derived from in vitro and animal studies or from small clinical cohorts. Larger, controlled trials are necessary to validate these results.

## 2. Materials and Methods

### 2.1. Study Design and Setting

We performed a retrospective, multicenter, observational study including consecutive patients with fibrotic interstitial lung disease (ILD) who developed lung cancer. Cases were identified from the institutional electronic medical records of participating Italian pulmonology and oncology centers between 2013 and 2024. All data were anonymized prior to analysis. The study was conducted in accordance with the Declaration of Helsinki and approved by the local ethics committees of the participating institutions. Patients involved provided written informed consent.

### 2.2. Eligibility Criteria

Patients were included if they met all of the following conditions:

1. Diagnosis of fibrotic ILD, confirmed by high-resolution computed tomography (HRCT) and/or histopathology, according to international consensus guidelines.
2. Diagnosis of primary lung cancer, confirmed by histology or cytology.
3. Availability of clinical and treatment data regarding both ILD and lung cancer.

Patients with pleural mesothelioma were identified but excluded from the main analyses, as mesothelioma is not considered a form of lung cancer and has distinct biological and therapeutic features.

Key exclusion criteria were insufficient clinical data or missing cancer diagnosis date.

### 2.3. Exposure Definition

The main exposure of interest was treatment with antifibrotic drugs (pirfenidone or nintedanib) prior to or at the time of lung cancer diagnosis. Of the 61 included patients, 35 had received antifibrotic therapy (19 with pirfenidone and 16 with nintedanib), while 26 had never been treated with antifibrotics.

- Antifibrotic group (AF): patients who had received antifibrotic therapy.
- No-antifibrotic group (NO): patients who had never received antifibrotic therapy.

### 2.4. Outcomes

The following outcomes were evaluated:

- Primary outcome: occurrence of acute exacerbation of ILD (AE-ILD) after the initiation of first-line oncologic therapy. AE-ILD was defined according to international consensus criteria as: (i) acute worsening of dyspnea within 30 days, (ii) new bilateral ground-glass opacities or consolidations superimposed on a background of fibrotic ILD on HRCT, and (iii) exclusion of alternative causes such as infection, pulmonary embolism, or heart failure.
- Secondary outcomes:
  1. PD-L1 expression in tumor tissue, recorded when available. Expression was categorized using the standard thresholds of ≥1% and ≥50% tumor proportion score.
  2. Autoimmunity was assessed using standard serological panels, including antinuclear antibodies (ANA), extractable nuclear antigen antibodies (ENA), antineutrophil cytoplasmic antibodies (ANCA), and rheumatoid factor. Results were categorized as positive or negative according to established laboratory cut-off values.
- Exploratory outcome: survival, expressed in months from cancer diagnosis to death. Importantly, survival time was available only for deceased patients, whereas for survivors the database recorded “alive” without a date of last contact. As a result, Kaplan–Meier survival curves and log-rank tests were not feasible, and survival is presented descriptively.

### 2.5. Oncologic Treatment Categories

First-line cancer treatments were categorized into four groups:

1. Surgery (S)
2. Chemotherapy (CHT)
3. Radiotherapy (RT)
4. Immunotherapy (IC)

Patients who received combined treatments were assigned to both categories.

### 2.6. Data Collection and Management of Missing Data

Clinical, demographic, functional, radiologic, and oncologic data were extracted retrospectively from electronic health records. The dataset included information on age, sex, smoking history, ILD type, antifibrotic exposure, cancer histology, treatment modality, AE-ILD occurrence, PD-L1 status, autoimmunity, and survival.

Missing data were not imputed. Analyses were conducted on a complete-case basis, and denominators were explicitly reported for each variable.

### 2.7. Statistical Analysis

Continuous variables were summarized as mean ± standard deviation (SD) or median and interquartile range (IQR), depending on distribution. Categorical variables were expressed as absolute counts and percentages.

- Group comparisons:
  ○ Binary outcomes (e.g., AE-ILD yes/no) between AF and NO groups were compared using Fisher’s exact test, given the limited sample size.
  ○ Continuous baseline variables were compared in terms of variance using Levene’s test (median-centered) to assess the equality of variances between AF and NO groups.
  ○ No formal between-group mean comparison was attempted for continuous variables with high missingness or small denominators.
- Subgroup analyses: exploratory and descriptive, stratified by first-line oncologic treatment, PD-L1 status, and autoimmunity. Given small subgroup sizes, no inferential statistics were applied, and results were interpreted cautiously.
- Survival: survival times were recorded numerically only for deceased patients. As censoring dates for survivors were systematically missing, Kaplan–Meier curves, median overall survival estimates, and log-rank comparisons could not be performed. Descriptive statistics (median survival among deceased patients) were reported, but no inferential time-to-event methods were applied.
- Software: All analyses were performed using JMP^®^ (v18.2.2, SAS Institute, Cary, NC, USA) and Jamovi (version 2.5). A two-sided *p* < 0.05 was considered statistically significant.
- Baseline variance analysis: Levene’s tests showed no evidence of unequal variances between antifibrotic (AF) and no-antifibrotic (NO) groups for BMI (*p* = 0.988), pack-years (*p* = 0.619), or DLCO% (*p* = 0.367). Age at ILD diagnosis showed a borderline, non-significant difference (*p* = 0.077). These findings suggest overall comparability of variance between groups, supporting the validity of subsequent group comparisons.
- Advanced statistical analyses were performed using the partitioning algorithm in the JMP software (JMP-Statistical Discoveries, SAS, www.jmp.com). Decision trees were employed as a classification method, using a series of hierarchical variable selections structured in a tree-like model. Variables (branches) were defined as splitting criteria.

Data were partitioned in a top-down iterative manner, progressively removing less relevant variables based on these criteria. The classifier recalculated variable significance at each iteration.

The decision tree thus represented a hierarchical partition of training examples, akin to a top-down clustering algorithm, though supervised by the class labels of the training instances.

Splitting predictors could be continuous or categorical (nominal or ordinal). For continuous predictors, cut-off values were used to dichotomize the sample. For categorical predictors, binary splits were applied.

For continuous outcomes, response means were calculated. For categorical outcomes, class probabilities were computed.

Uncertainty in classification was assessed using several numerical indices, including the Gini index (IG) and entropy (H). These indices informed the choice of partitions during tree construction.

Statistical plots were also generated using the online Jamovi open-source platform.

### 2.8. Limitations

An important limitation of our study is the heterogeneity of the population, which included multiple lung cancer histologies and lacked systematic information on tumor stage. Moreover, survival times were recorded only for deceased patients, whereas censoring dates for survivors were missing. As a result, robust survival analyses (Kaplan–Meier curves, log-rank tests, or stage-stratified comparisons) could not be performed, and survival outcomes must be interpreted only in a descriptive, hypothesis-generating way.

## 3. Results

### 3.1. Patient Characteristics

The study population included 46 males and 15 females. Regarding smoking history, 14 were current smokers, 32 former smokers, and 15 never-smokers.

ILD diagnoses were as follows: 43 patients with idiopathic pulmonary fibrosis (IPF), 6 with combined pulmonary fibrosis and emphysema (CPFE), 6 with fibrosing NSIP, 1 with fibrosing hypersensitivity pneumonitis (HP), 1 with sarcoidosis, 2 with CTD-associated UIP, 1 with unclassifiable ILD, and 1 with non-IPF UIP.

Among the 35 patients receiving antifibrotic therapy, 19 were treated with pirfenidone and 16 with nintedanib.

Regarding tumor histology, 38 patients had adenocarcinoma, 13 squamous cell carcinoma (SCC), 6 SCLC, 3 undifferentiated carcinoma, and 1 pleural mesothelioma (excluded from analysis).

Although limited by a small sample size, our findings confirmed that SCC is more common in CPFE than in other ILDs or the general population (SCC 33.3%, adenocarcinoma 33.3%).

Survival was poorer in patients with CPFE compared to other ILD subtypes: mean survival after cancer diagnosis was 10.2 months in CPFE, 49.1 months in CTD-ILDs, and 20.9 months in IPF.

First-line cancer treatment (according to national guidelines) included: surgical resection in 12 cases, chemotherapy alone in 20, radiotherapy alone in 7, chemoradiotherapy in 3, immune checkpoint inhibitors in 6, and no treatment in 13.

### 3.2. Survival and AE-ILD

**Figure 1:**
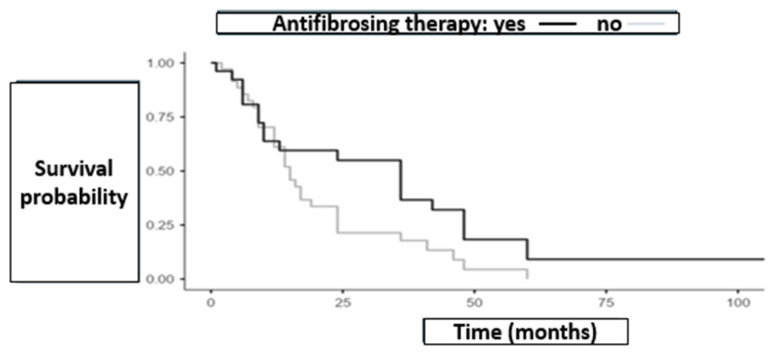
Illustrates the survival curves over time for the two groups (antifibrotic therapy group = 35, non-antifibrotic therapy group = 26). Curves are descriptive only as no time-to-event analysis were feasible due to missing censoring data.

Survival data were available only for deceased patients, whereas censoring dates for survivors were systematically missing. As a result, Kaplan–Meier curves, median OS estimates, and log-rank tests could not be performed. Reported mean OS values therefore represent descriptive measures among deceased patients only and should not be interpreted as inferential comparisons. In this context, mean OS was 17.9 months in the antifibrotic group and 33.2 months in the non-antifibrotic group. Figure 1 illustrates the survival curves over time for the two groups.

The longer survival observed in the non-antifibrotic group is likely attributable to the higher proportion of patients with CTD-ILD, a condition with a significantly better prognosis compared to IPF and CPFE, which were the predominant diagnoses among patients on antifibrotic therapy.

Pulmonary function tests at the time of ILD diagnosis further support this interpretation, showing milder disease in the non-antifibrotic group (mean FVC: 2.81 L vs. 2.67 L; mean DLCO: 61.92% vs. 52.75%, respectively).

Despite the increased risk associated with anticancer treatment, only 7 patients in our cohort developed acute exacerbation of ILD (AE-ILD); 6 had IPF and 1 had sarcoidosis.

Among them, 4 cases occurred in the antifibrotic group (11.4% of patients receiving antifibrotic therapy) and 3 in the group not receiving antifibrotics or treated with immunosuppressive therapy (11.5% of patients not on antifibrotics).

**Figure 2:**
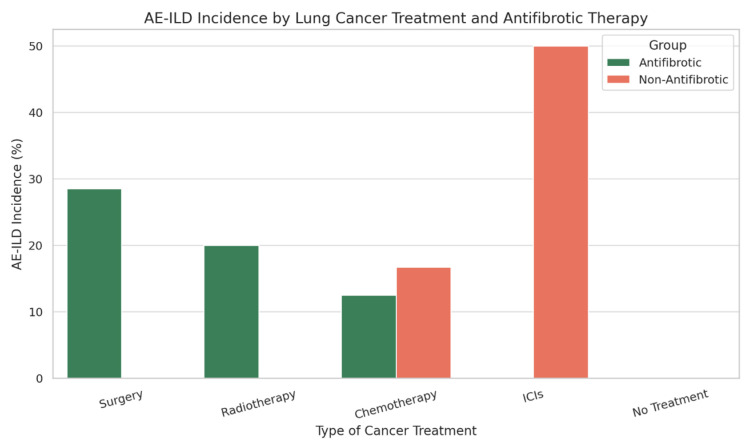
Incidence of AE-ILD stratified by type of cancer treatment and antifibrotic therapy status (35 patients received antifibrotic therapy with 4 AE-ILD, 26 not received antifibrotic therapy with 3 AE-ILD). Abbreviations: Antifibrotic = antifibrotic therapy; Non-antifibrotic = non-antifibrotic therapy; AE-ILD = acute exacerbation of intestitial lung disease; ICIs = immune checkpoint inhibitors.

When stratified by type of lung cancer treatment, AE-ILD occurred in antifibrotic vs. non-antifibrotic groups as follows: 28.5% vs. 0% in patients undergoing surgery; 20% vs. 0% in those receiving radiotherapy; 12.5% vs. 16.7% in patients treated with chemotherapy; 0% vs. 50% in those treated with immune checkpoint inhibitors (ICIs); and no cases in patients who did not undergo cancer treatment in either group. (Figure 2 and Table 1)

**Figure 3:**
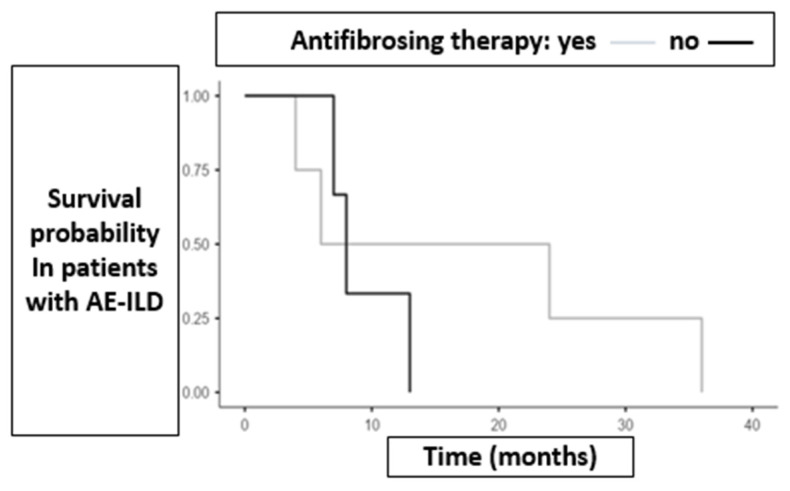
Illustrates survival outcomes in both groups of patients who experienced AE-ILD. Antifibrotic group *n* = 4, non-antifibrotic group = 3. Curves are descriptive only as no time-to-event analysis were feasible due to missing censoring data. Abbreviations: AF = antifibrotic therapy; NO = non-antifibrotic therapy; ILD = interstitial lung disease.

Mean survival among patients who developed AE-ILD was higher in the antifibrotic group compared to the non-antifibrotic group (17.5 months vs. 9.33 months). Figure 3 illustrates survival outcomes in both groups of patients who experienced AE-ILD.

No significant differences were observed in relation to pulmonary function parameters. Contrary to what has been reported in the literature, in our cohort a reduced DLCO did not correlate with an increased risk of AE-ILD (mean DLCO in patients who developed AE was 63% vs. 56% in those who did not). The same was observed for FVC, with a mean value of 2.84 L in AE patients vs. 2.73 L in those without AE.

Three additional parameters were analyzed as potential risk factors for AE: BMI (mean BMI 26.21 in patients with AE vs. 27.35 in those without), age at ILD diagnosis (mean age 71.4 years vs. 69 years), and age at lung cancer diagnosis (mean age 74 years vs. 71.5 years, respectively).

Among never-smokers, 20% developed AE-ILD. In contrast, only 4 out of 46 patients with a current or past smoking history developed AE-ILD (8.7%). Furthermore, the incidence of AE-ILD was higher in former smokers than in current smokers (6.5% vs. 2.2%).

### 3.3. Role of Antifibrotic Therapy in the Timing of Lung Cancer Onset

In the group of patients receiving antifibrotic therapy, the mean time interval between ILD diagnosis and lung cancer diagnosis was 2 years. The majority of patients in this group had a diagnosis of IPF or CPFE.

In the group of patients not receiving antifibrotic therapy, we distinguished between those who had never received any treatment and those who had been treated with or were undergoing immunosuppressive therapy. In the latter subgroup, the most frequent ILDs were CTD-ILD (6 out of 10 cases), followed by 1 case each of HP, sarcoidosis, CPFE, and unclassifiable ILD. Since IPF was excluded and only one case of CPFE was present, the mean time between ILD and cancer diagnosis was 4.2 years.

**Figure 4:**
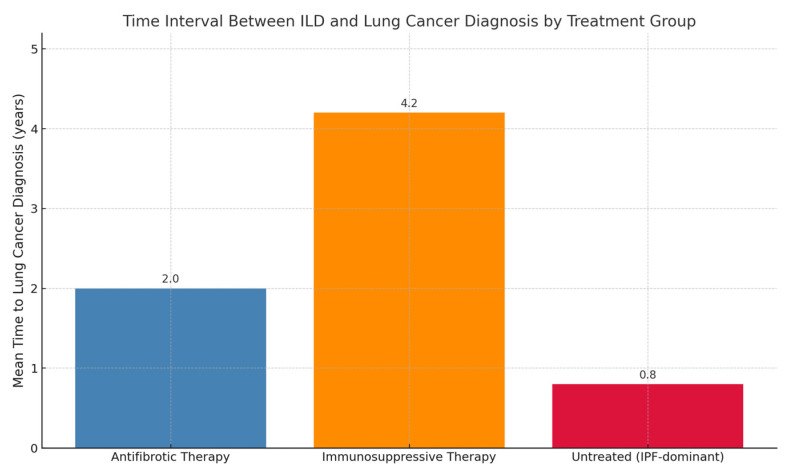
Illustrating the mean interval time between ILD and lung cancer diagnosis across different treatment groups. Antifibrotic therapy group *n* = 35, non-antifibrotic therapy group *n* = 26 (10 on immunosuppressive therapy, 16 untreated).

In the untreated group (no antifibrotics nor immunosuppressants), most patients had IPF—specifically, 12 out of 16. However, data on the ILD diagnosis year were missing for 2 patients and thus excluded from the statistical analysis. This subgroup was more comparable in ILD phenotype to the antifibrotic group. When comparing the untreated group to the antifibrotic group, survival was shorter in the untreated cohort (0.8 years vs. 2 years) (Figure 4).

### 3.4. Effects of Antifibrotic Therapy According to Tumor Histology

In the adenocarcinoma (ADK) subgroup, mean survival was nearly identical between patients receiving antifibrotic therapy and those who did not (15.8 vs. 16.6 months, respectively).

In contrast, among other histological subtypes, a more pronounced difference in survival was observed in favor of patients treated with antifibrotics.

In squamous cell carcinoma (SCC), patients on antifibrotic therapy had a mean survival of 15.8 months compared to 7 months in those not receiving such therapy. A similar trend was observed in small-cell lung cancer (SCLC), with a mean survival of 21.5 months in the antifibrotic group vs. 6.7 months in the non-antifibrotic group. These findings should be considered descriptive and hypothesis-generating only, as no time-to-event analyses were feasible due to incomplete survival data.

**Figure 5:**
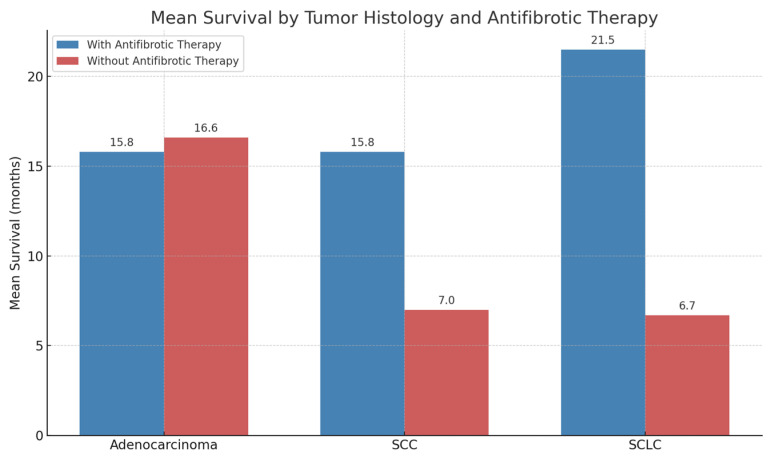
Mean survival by tumor histology in patients with and without antifibrotic therapy. Adenocarcinoma *n* = 38, squamous cell carcinoma *n* = 13, small-cell lung cancer *n* = 6. Curves are descriptive only as no time-to-event analysis were feasible due to missing censoring data. Abbreviations: SCC = squamous cell carcinoma; SCLC = small-cell lung cancer.

The figure below illustrate survival by tumor histology in patients receiving antifibrotic therapy and those not receiving antifibrotic therapy (Figure 5).

### 3.5. Role of PD-L1

**Figure 6:**
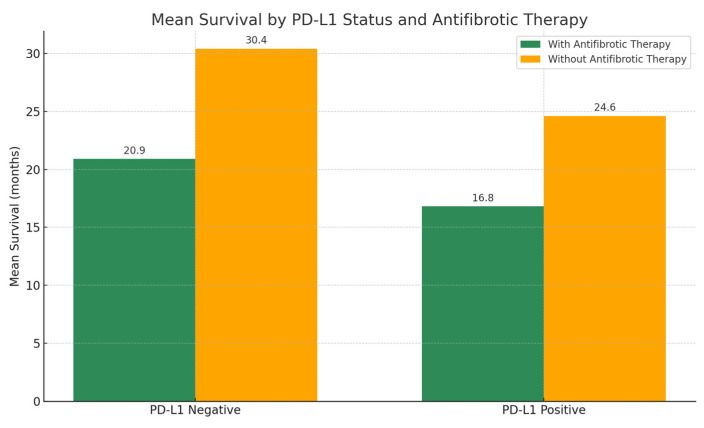
Mean survival by PD-L1 status in patients with and without antifibrotic therapy. Antifibrotic therapy group *n* = 35, no antifibrotic therapy group *n* = 26. Curves are descriptive only as no time-to-event analysis were feasible due to missing censoring data.

The mean expression of PD-L1 in tumor cells was 24.1% in the group receiving antifibrotic therapy and 21.8% in the group not receiving antifibrotic treatment. Among patients treated with antifibrotics, those whose tumors did not express PD-L1 showed longer mean survival compared to those with PD-L1 expression (20.9 vs. 16.8 months, respectively). A similar trend was observed in the non-antifibrotic group, where mean survival was 30.4 months in patients without PD-L1 expression and 24.6 months in those with PD-L1-positive tumors (Figure 6).

Interestingly, all cases of AE-ILD in the antifibrotic group occurred in patients with PD-L1–negative tumors. Conversely, in the non-antifibrotic group, AE-ILDs were observed exclusively in patients with PD-L1–positive tumors.

### 3.6. Role of Autoimmunity

Autoantibody positivity was observed in 16 out of 61 patients (26.2%). The proportion was higher in the group not receiving antifibrotic therapy (30.7%) compared to the antifibrotic group (22.8%). This difference is likely attributable to the fact that a substantial portion of patients not treated with antifibrotics had connective tissue disease-associated interstitial lung disease (CTD-ILD), and within our cohort, none of these patients were receiving antifibrotic agents. Instead, they were treated exclusively with immunosuppressive therapies (steroids and methotrexate).

As a result, the difference in survival among patients with positive autoimmunity between the antifibrotic and non-antifibrotic groups was considerable (13.9 vs. 48.1 months, respectively). This survival gap is largely explained by the underlying ILD phenotype: patients not on antifibrotics had CTD-ILD, a condition with a more favorable prognosis compared to idiopathic pulmonary fibrosis (IPF), which was the most represented diagnosis among those receiving antifibrotic treatment.

Among patients who were autoantibody-negative, mean survival was similar between the two groups (19.5 months with antifibrotics vs. 22.6 months without). Notably, within the antifibrotic group, patients with positive autoimmunity had shorter survival compared to those who were seronegative (13.9 vs. 19.5 months). However, none of the patients with autoantibody positivity developed AE-ILD, regardless of antifibrotic treatment status. The observed differences in autoantibody prevalence between groups are most likely attributable to the distribution of underlying ILD subtypes (with CTD-ILD cases being more frequent in the non-antifibrotic group) rather than any direct effect of antifibrotic therapy.

## 4. Discussion

This multicenter study suggests that, in a heterogeneous real-world cohort of fibrotic ILD with lung cancer, antifibrotic therapy was not clearly associated with differences in post-treatment AE-ILD rates or OS after adjustment. Given sample size and heterogeneity, these findings should be interpreted as descriptive and hypothesis-generating.

### 4.1. Clinical Implications

Continuation of antifibrotics during cancer care may be considered on a case-by-case basis when tolerated, as we did not observe a signal for increased AE-ILD risk; however, individualized risk assessment remains essential, particularly around chemotherapy, radiotherapy, and immune checkpoint inhibitors. PD-L1 status and autoimmune features showed exploratory associations only and should not currently guide management in this population.

### 4.2. Limitations

The retrospective design entails selection bias and residual confounding. The cohort is small (*n* = 61) and includes diverse ILD subtypes and tumor histologies/stages, limiting power—especially for subgroup analyses. Missing data further reduce precision; we used available-case analyses without imputation. Finally, the study was not designed to infer causality, and any temporal patterns (e.g., suggestion that antifibrotics might delay cancer onset) are speculative and require prospective validation.

Patients with fibrosing ILDs exhibit a significantly increased risk of developing lung cancer, which translates into higher overall mortality. The coexistence of these two conditions leads to a worsening of prognosis, and treatment of lung cancer introduces additional risks in individuals with underlying pulmonary fibrosis. The overall survival in our cohort was lower compared to patients with fibrosing ILD alone—25.5 months vs. the 3–5 years generally reported in the literature—partly due to the neoplastic overlap.

Acute exacerbations (AE-ILD) remain a relevant cause of mortality, and their risk increases following surgical, chemotherapeutic, or radiotherapeutic interventions. Although antifibrotic therapy appears to exert a protective effect against exacerbations, data from our cohort did not reveal significant differences in the incidence of tumor treatment-related AE-ILD between patients receiving antifibrotics and those who were not (11.4% vs. 11.5%). Similarly, subgroup analysis based on type of oncologic treatment did not reveal substantial differences, likely due to the small sample size.

Previous studies, such as that by Otsubo et al., have shown that careful patient selection—based, for example, on GAP stage (stage I or II)—may help reduce the risk of AE-ILD in patients undergoing oncologic therapies. In our population, GAP staging was not assessed; however, analysis of parameters such as age at ILD diagnosis and baseline lung function did not reveal significant differences between patients with and without AE-ILD. Conversely, older age at cancer diagnosis was associated, albeit not significantly, with a higher risk of AE-ILD. Notably, the proportion of AE-ILD events was higher among never-smokers compared to current or former smokers.

Although the incidence of AE-ILD was not statistically significant, mean survival was longer in the group receiving antifibrotic therapy, including among patients who experienced AE-ILD.

In our cohort, patients receiving antifibrotics showed a numerically longer interval between ILD and lung cancer diagnosis compared to untreated patients (0.8 vs. 2 years). This observation is hypothesis-generating and should be interpreted with caution, as causality cannot be inferred from retrospective data. However, it remains unclear whether this effect is directly attributable to the action of antifibrotic agents on ILD progression or on tumor development. Obviously, this hypothesis needs further and deeper validation.

Regarding PD-L1 expression in tumor cells, no significant differences were found between the two groups. Nevertheless, a more in-depth analysis suggested a possible protective role of PD-L1 in reducing AE-ILD risk among patients receiving antifibrotic therapy, a phenomenon not observed in patients without such treatment. Additionally, the non-antifibrotic group had a higher prevalence of autoantibody positivity, likely due to the predominance of CTD-ILD cases treated with immunosuppressive agents, which are known to confer better survival compared to IPF.

Interactions between antifibrotics and immune checkpoint inhibitors (ICIs) are still poorly understood, particularly with regard to their potential protective role against AE-ILD and ICI-induced pulmonary toxicity.

Although their protective role against cancer therapy-related AE-ILD requires further investigation, preliminary evidence has shown encouraging results. Moreover, due to the shared pathogenic mechanisms between ILD and lung cancer, a potential antineoplastic activity of antifibrotics has been hypothesized.

The data obtained suggest several important directions for future research. First, it will be crucial to deepen the molecular characterization of lung cancers associated with ILD, exploring the potential impact of antifibrotic agents on specific genetic mutations. A more comprehensive understanding of these mechanisms may open new avenues for the development of targeted therapies, improving the integrated management of both conditions. Furthermore, the overlap in pathogenic pathways between ILD and lung cancer presents an opportunity to identify novel shared therapeutic targets. Future studies could focus on these common molecular pathways to develop treatments capable of limiting both fibrotic progression and neoplastic growth.

Our study underscores the importance of antifibrotic therapy in patients with concomitant ILD and lung cancer, demonstrating improved survival without a significant increase in toxicity or adverse events. Despite the study’s limitations, the results provide a solid foundation for further research, contributing to a better understanding of the complex interactions between ILD, lung cancer, and innovative treatments. The relevance of these findings lies in their potential to guide future clinical strategies toward a more personalized and multidisciplinary approach to these challenging diseases.

## 5. Conclusions

Our multicenter retrospective study provides novel insights into the interplay between antifibrotic therapy and oncologic outcomes in patients with fibrotic interstitial lung disease (ILD) who develop lung cancer, a complex and increasingly encountered clinical scenario.

From a clinical standpoint, our data suggest several practical considerations for physicians managing patients with fibrotic ILD who develop lung cancer. First, continuation of antifibrotic therapy during oncologic treatment appears feasible, as antifibrotics were not associated with an increased risk of AE-ILD in our cohort. Although a protective effect could not be demonstrated, the absence of a clear safety signal supports individualized continuation rather than discontinuation, particularly in patients with progressive ILD.

Second, the finding that nearly half of evaluable tumors expressed PD-L1 ≥ s1% (and approximately one-quarter ≥50%) underscores that a substantial proportion of this population may be candidates for immune checkpoint inhibitors. However, given the known risk of immunotherapy-related pneumonitis and the vulnerability of fibrotic lungs, multidisciplinary risk–benefit assessment is mandatory. Antifibrotic therapy should not be considered a contraindication to immunotherapy, but heightened vigilance for respiratory toxicity is advised.

Third, autoimmunity was present in about one-quarter of patients, which is relevant both diagnostically (as ILD may have autoimmune features) and therapeutically (given the potential for enhanced toxicity under immunotherapy). In this setting, autoimmune positivity should not automatically preclude oncologic treatment, but clinicians should involve rheumatology specialists and closely monitor for exacerbation or immune-related adverse events.

Taken together, our findings highlight that management of lung cancer in fibrotic ILD requires personalized, multidisciplinary decision-making. Until larger prospective data are available, antifibrotics can generally be maintained, PD-L1 status should inform immunotherapy eligibility with caution, and autoimmunity should prompt closer surveillance rather than outright exclusion from oncologic care.

In conclusion, in our multicenter retrospective study, antifibrotic therapy was not associated with an increased risk of AE-ILD following cancer treatment in patients with fibrotic ILD and lung cancer. Approximately half of evaluable tumors expressed PD-L1, and autoimmunity was present in about one-quarter of patients, both potentially relevant for treatment decision-making. However, the heterogeneity of tumor histologies, lack of systematic staging, and incomplete survival data preclude definitive conclusions regarding survival outcomes. Our findings should therefore be viewed as exploratory and hypothesis-generating, emphasizing the need for larger, prospective studies with standardized data collection.

## Figures and Tables

**Table 1:** Incidence of Acute exacerbation of ILD (AE-ILD) by cancer treatment type and antifibrotic therapy. Abbreviations: AE-ILD = acute exacerbation of interstitial lung disease.

Cancer Treatment Type	AE-ILD Incidence (%)—Antifibrotic Group	AE-ILD Incidence (%)—Non-Antifibrotic Group
Surgery	28.5%	0%
Radiotherapy	20%	0%
Chemotherapy	12.5%	16.7%
Immune Checkpoint Inhibitors (ICIs)	0%	50%
No Cancer Treatment	0%	0%

## Data Availability

The data generated and analyzed during the current study are not publicly available due to privacy concerns but are available from the corresponding author on reasonable request.
